# Hypotension prediction index to predict hemodynamic instability related to renal replacement therapy in critically ill patients: A prospective observational study

**DOI:** 10.1016/j.jointm.2026.03.002

**Published:** 2026-05-09

**Authors:** Céline Monard, Clemence Ferlay, Livia Whiting, Bastien Trächsel, Antoine Schneider

**Affiliations:** 1 Adult Intensive Care Unit, Centre Hospitalier Universitaire Vaudois (CHUV), Lausanne, Switzerland; 2 Faculty of Biology and Medicine (FBM), University of Lausanne (UNIL), Lausanne, Switzerland; 3 Department of Epidemiology and Health Systems, Centre for Primary Care and Public Health (Unisanté), University of Lausanne, Lausanne, Switzerland

**Keywords:** Renal replacement therapy, Fluid removal, Hypotension, Prediction, Hypotension prediction index

## Abstract

**Background:**

Fluid removal during continuous renal replacement therapy (CRRT) is a frequent cause of hemodynamic instability (HI) in critically ill patients, yet clinicians lack reliable tools to anticipate intolerance to ultrafiltration. We aimed to determine the performance and clinical utility of the hypotension prediction index (HPI) to predict HI in critically ill patients undergoing fluid removal with CRRT.

**Methods:**

We conducted a prospective observational study including consecutive patients receiving CRRT with fluid removal in a tertiary intensive care unit (ICU) of Centre Hospitalier Universitaire Vaudois, Lausanne, Switzerland, between March 2022 and August 2023. Arterial pressure waveforms were recorded using FloTrac and HemoSphere Advanced Monitor and HPI was computed every 20 s (offline, for blinding). An HPI alarm was defined as three consecutive HPI values ≥85; an HI event was defined as three consecutive mean arterial pressure readings <65 mmHg, initiation or escalation of vasopressor therapy, or clinical signs of hemodynamic intolerance. We evaluated the performance of HPI for predicting HI within the following 15-min window. To assess HPI clinical utility at bedside, we computed alarm-to-event delays, the proportion of HPI alarms followed by HI and the proportion of HI preceded by at least one HPI alarm. We repeated analyses considering only “new” alarms and “new” HI, defined respectively as alarms and HI events preceded by a 60-min event-free interval.

**Results:**

We recorded 223,660 observations corresponding to 1243 h of monitoring. HPI was ≥85 in 33.4% of observations and MAP <65 mmHg in 11.0%. Overall, HPI demonstrated good discriminative performance for predicting HI (area under the curve of the receiver operating characteristic curve=0.89), with high sensitivity (99.7%) and specificity (81.6%). However, positive predictive value (PPV) was low, as only 54.7% of alarms were followed by HI, with a mean alarm-to-event delay of 103 s. Bootstrapped analyses performed to adjust for varying importance of individual patients confirmed these findings. When considering new alarms (*n*=147) and events (*n*=174), only 33 (22.4%) alarms were followed by an HI, and 34 (19.4%) HI were preceded by an alarm. Sensitivity analyses considering a lower cutoff for HPI and a shorter event-free period to define a new event confirmed these findings.

**Conclusions:**

Although HPI shows excellent statistical performance for predicting HI during CRRT with fluid removal, its clinical utility is limited by short time-to-event delay, frequent false-positive alarms, and prolonged time with high HPI values. These findings underscore the gap between predictive accuracy and bedside utility of monitoring tools in critical care.

## Introduction

Fluid overload is a major concern in critically ill patients, particularly those with severe acute kidney injury (AKI) and oliguria.^[^^1^^]^ It is associated with prolonged intensive care unit (ICU) and hospital stays, compromised renal recovery, and increased mortality.^[^[2], [3], [4]^]^ To improve outcomes, authors have suggested that fluid removal should be undertaken as soon as possible after the initial resuscitation phase.^[^^5^^]^ In patients receiving continuous renal replacement therapy (CRRT), rapid fluid removal can be achieved by plasma ultrafiltration (net ultrafiltration). To date, the ideal net ultrafiltration rate for a given patient remains difficult to establish, as too intense removal may lead to hemodynamic instability (HI) and organ dysfunction, while too slow removal may prolong mechanical ventilation and ICU stay.^[^[6], [7], [8]^]^ Current approach typically includes a low (50 mL/h) initial net ultrafiltration with progressive increase depending on tolerance. Net ultrafiltration is stopped or reduced as a response to hypotension, tachycardia, mottling or increased blood lactate levels.^[^^9^^]^ However, this pragmatic approach is user-dependent and may lead to repeated episodes of hypoperfusion, which could affect renal recovery.^[^^10^^]^ A tool guiding fluid removal during CRRT would be desirable and could improve the safety and efficiency of fluid removal.^[^^11^^,^^12^^]^

The hypotension prediction index (HPI) algorithm was recently developed using artificial intelligence and machine learning models to predict the likelihood of a patient experiencing a hypotensive event.^[^^13^^]^ Based on arterial pressure curve characteristics and calculated advanced hemodynamic parameters, it generates a score (the HPI value, displayed on a monitor) ranging from 1 to 100. The HPI value indicates the probability of hypotension (defined by a mean arterial pressure [MAP] <65 mmHg) in the following minutes. While evaluated in surgical settings with promising results,^[^[14], [15], [16]^]^ its performance and usefulness in other settings, such as during fluid removal in critically ill patients, where numerous other confounding factors could influence its prediction, remains unknown.

Accordingly, we designed a prospective observational study aiming to assess the discriminative properties and clinical utility of HPI to predict HI episodes in critically ill patients undergoing fluid removal with CRRT. Our primary objective was to determine the proportion of HPI alarms that correctly predicted an HI as a measure of the test’s diagnostic accuracy and clinical utility.

## Methods

### Study design and population

This prospective observational single-center study was conducted in the Centre Hospitalier Universitaire Vaudois (CHUV), Lausanne, Switzerland, between March 2022 and August 2023. Consecutive adult patients (≥18 years old) admitted to the ICU, with a functioning radial arterial line and receiving CRRT with fluid removal (net ultrafiltration rate >0 mL/h), were eligible for inclusion. Patients with a prescribed MAP target >65 mmHg (e.g., ≥70–75 mmHg in specific clinical contexts such as brain injury or chronic hypertension), as well as those with cardiac arrhythmias, severe cardiac shunts, severe aortic stenosis, receiving extracorporeal membrane oxygenation, or enrolled in a conflicting trial, were excluded.

### CRRT protocol

All patients underwent CRRT according to our local protocol.^[^^17^^]^ Therapies were run in continuous veno-venous hemodialysis mode (CVVHD) with standard fluid solutions. Dialysate flow rate was set according to patients’ dry weight, aiming to deliver an effluent rate between 20 and 25 mL/(kg·h), in accordance with Kidney Disease: Improving Global Outcomes (KDIGO) guidelines. Anticoagulation of the circuit was either regional with citrate-calcium or systemic with heparin. During CRRT, target MAP was set at 65 mmHg. Fluid removal rate, hemodynamic management, use of vasopressors, and fluid administration were left to the discretion of the clinical team.

### Data collection and definitions

We collected patient’s baseline, sociodemographic characteristics as well as biological and clinical parameters throughout the observation period from electronic medical records (Metavision [IMD Soft, Tel Aviv, Israel] and Soarian [Cerner, North Kansas City, USA]).

For each patient enrolled, a FloTrac device was added to the radial arterial line and connected to an HemoSphere Advanced Monitor (Edwards Lifesciences, Irvine, CA) to enable arterial waveforms recording. Blinding was achieved by setting the monitor to display only a limited number of values (systolic, diastolic, MAP, heart rate), but not the HPI value.

The total observation period corresponded to the time during which the FloTrac device was in place and was aimed to last for a minimum of 24 h per patient. No maximal recording duration was set. Periods during which CRRT or fluid removal was interrupted, cardiac rhythm was atrial fibrillation, or technical issues compromised arterial waveform recording were excluded from HPI evaluation.

At the end of the study, arterial waveforms were downloaded from the HemoSphere Advanced Monitors, and the HPI algorithm (Edwards Lifesciences, Irvine, CA) was applied offline. The algorithm generates an HPI value every 20 s. Thus, the final dataset contained single observations (HPI and hemodynamic values, including systolic and diastolic arterial pressure, MAP, heart rate) every 20 s. If the interval between two consecutive observations exceeded 60 s, additional rows of missing values were inserted every 20 s to maintain uniform temporal resolution.

In the main analysis, an “HPI alarm” was defined as three consecutive HPI values ≥ 85 within 1 min, indicating a high probability of hypotension within the subsequent 15 min. An episode of HI was defined by the occurrence of one of the following events: (1) actual hypotension defined by three consecutive values of MAP <65 mmHg within 1 min, (2) the need to start norepinephrine infusion to reach a MAP of 65 mmHg, (3) a significant increase in the rate of norepinephrine (>5 µg/min within 30 min), and (4) a clinical sign of hemodynamic intolerance to net ultrafiltration, including new-onset atrial fibrillation, hyperlactatemia, sinus tachycardia, low cardiac output, and cutaneous mottling.

To differentiate new events from sustained ones, we repeated analyses considering only acute changes in HPI status or the patient’s hemodynamic situation. Here, “new” HPI alarm or “new” HI were defined as an HPI alarm or HI preceded by a 60-min event-free period (Supplementary Figure S1). An explanation of the choice of the event-free period duration is provided in the statistical analyses section.

### Ethics

This study was conducted in accordance with the *Declaration of Helsinki*, the Human Research Act and the Human Research Ordinance.^[^^18^^,^^19^^]^ The study protocol as well as the consent procedure were approved by the Ethics Committee of Vaud (2022-00065). As this study involved patients in an emergency situation, written informed consent was obtained from each patient or their next of kin before enrolment. In all cases, the patient’s consent was subsequently sought.

### Statistical analyses

In the absence of data obtained in the context of fluid removal during CRRT at the time of study preparation, a sample size could not be calculated *a priori*. We considered 40 patients observed over a prolonged period as a convenience sample. All data were prospectively collected and managed using REDCap electronic data capture tools hosted at our institution. Continuous data are reported as median (interquartile range, IQR), and categorical variables are expressed as number (percentage).

First, we quantified the incidence of HPI alarms and HI both across the entire dataset and at the individual patient level. We assessed the discriminative performance of continuous HPI values for predicting HI within a 15-min forecasting window; this was assessed by the area under the receiver operating characteristic curve (AUROC), with adjustment for repeated measures.^[^^20^^]^ We also computed the area under the precision recall curve (AUPRC) which is more robust in the context of high class imbalance. Then, using the HPI threshold recommended by the manufacturer (85), we calculated the sensitivity, specificity, positive predictive value (PPV), and negative predictive value (NPV) of an HPI alarm to predict HI within 15 min. A true positive (TP) was defined as an HPI alarm followed by at least one HI within 15 min. A false positive (FP) was defined as an HPI alarm not followed by an HI within 15 min. A false negative (FN) was defined as an HI not preceded by any HPI alarm in the previous 15 min. A true negative (TN) was defined as a time point with three consecutive values of HPI <85 and not followed by any HI within 15 min. PPV was defined as the proportion of HPI alarms that correctly predicted an HI (=TP/[TP + FP]), and sensitivity was defined as the proportion of HI episodes that were correctly predicted by a preceding HPI alarm (TP/[TP + FN]).

To account for variable observation durations across patients and to prevent overrepresentation of individuals with longer follow-up while appropriately addressing within-patient correlation, we conducted patient-level bootstrap resampling for repeated measures, treating each patient as the sampling unit and resampling an equal number of observations per patient.

Then, to assess whether HPI would be mirroring current MAP rather than predicting future MAP, as suggested in previous studies,^[^^21^^]^ we performed an association analysis between HPI and MAP values. For this purpose, we used a flexible model with random effect,^[^^22^^]^ accounting for repeated measurements among subjects to fit data distribution, and reported an *R*^2^.^[^^23^^,^^24^^]^ We assessed the association between HPI and MAP when measured either concomitantly or 15 min apart. We also computed the delay between each HPI alarm and the nearest subsequent HI event occurring within the 15-min prediction window.

To distinguish sustained from new events within our high-frequency dataset, we performed an autocorrelation test to determine the minimum temporal separation required between two events to assume their independence. We selected an autocorrelation threshold of 0.707, which is equivalent to an *R*^2^ of 50%, i.e., a model using the current value to predict the future value would explain 50% of the variability. Based on this analysis, the estimated temporal separation between two distinct events was 62 min for HPI alarms and 53 min for HI. Hence, we selected a common conservative threshold of 60 min to define two events as independent (e.g. an event-free period of 60 min was required to qualify an event as “new”).

New HPI alarms and new HI were characterized by their incidence rates across the entire cohort and at the individual patient level. We reported TP, TN, FP, and FN when considering only these new events. In these analyses, to prevent artificial inflation of TN counts, the analysis window was moved forward by a time interval equal to the event-free period (60 min) after each TN identification, ensuring that no two TNs could be assigned within <60 min of one another. We also reported the proportion of new HPI alarms followed by a new HI within 15 min, the proportion of new HI events preceded by at least one new HPI alarm within the preceding 15 min, and the alarm-to-event delays.

Finally, we performed sensitivity analyses where we repeated our main analysis considering another threshold to define an HPI alarm (75 instead of 85), and a shorter duration to define event independence (3-min free-of-event instead of the 60-min duration identified in the autocorrelation analysis).

Statistical analyses were performed with STATA version 17.0 (StataCorp LP, College Station, TX) and R version 4.4.3 (R Core Team 2025, https://cran.r-project.org/web/packages/report/vignettes/cite_packages.html). A two-tailed *P* value <0.05 was considered statistically significant.

## Results

### Population

During the study period, 117 patients were screened for eligibility and 41 met all inclusion criteria and were enrolled. Eleven patients (9.5%) were excluded solely due to the absence of net fluid removal (Figure 1). One patient withdrew consent and was excluded from analyses. As shown in Table 1, participants were mostly males (*n*=29, 72.5%) with a median Simplified Acute Physiology Score (SAPS)-II score of 46 (IQR: 38–55). The median delay between ICU admission and CRRT initiation was 47 (18–88) h. The median delay between CRRT initiation and recording was 67 (20–134) h. Twelve (30.0%) patients died in the ICU. At hospital discharge, among the 25 (62.5%) patients who survived, only 4 (16.0%) remained dialysis dependent.

**Figure 1:**
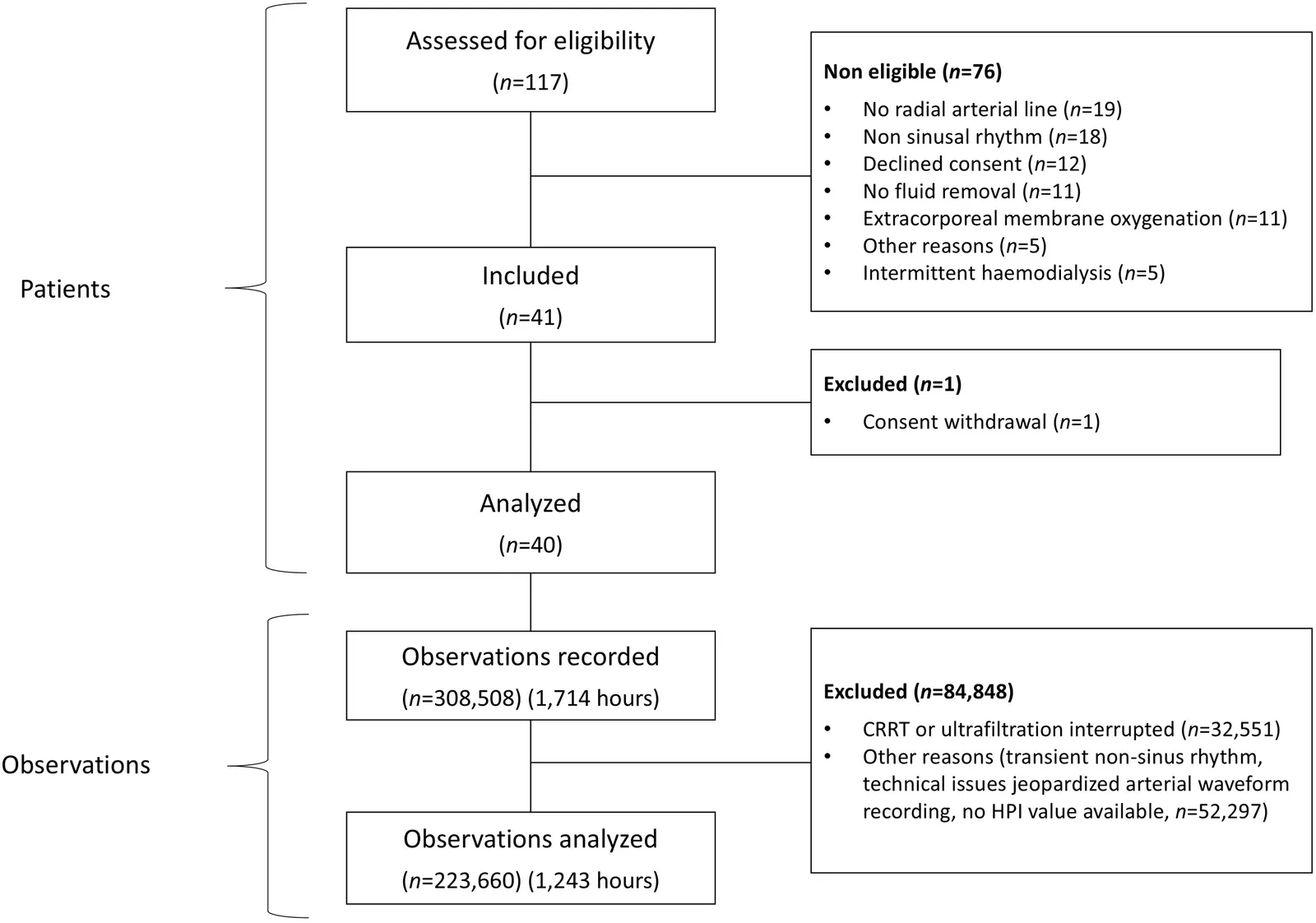
Flow chart of patients and observations selection. One observation is obtained every 20 s of recording. CRRT, Continuous renal replacement therapy; HPI, Hypotension prediction index.

**Table 1:** Basic characteristics of patients.

Characteristics	Data
Age (years)	66 (58–70)
Sex, male	29 (72.5)
Charlson Comorbidities Index	5 (3–6)
Chronic kidney disease	7 (17.5)
Hypertension	21 (52.5)
Characteristics at admission	
Medical admission	27 (67.5)
Surgical admission	13 (32.5)
SAPS–II score at ICU admission	46 (38–55)
Delay from ICU admission to CRRT initiation (hours)	47 (18–88)
Delay from CRRT initiation to recording beginning (hours)	67 (20–134)
Characteristics at the beginning of recording	
Cumulative fluid balance (L)	5.40 (1.40–9.20)
Invasive mechanical ventilation	33 (82.5)
Norepinephrine administration	18 (45.0)
pH	7.42 (7.38–7.46)
PaCO_2_ (mmHg)	38 (35–41)
Lactate (mmol/L)	1.30 (1.00–1.80)
Sodium (mmol/L)	138 (136–139)
Albumin (g/L)	28 (24–31)
Hematocrit(%)	25 (23–27)
SOFA score	12 (9–15)
Characteristics at the end of recording	
Cumulative fluid balance (L)	2.20 (−2.30 to 8.30)
Invasive mechanical ventilation	29 (72.5)
Norepinephrine administration	17 (42.5)
pH	7.45 (7.41–7.49)
PaCO_2_ (mmHg)	37 (34–40)
Lactate (mmol/L)	1.25 (1.00–1.70)
Sodium (mmol/L)	138 (137–139)
Albumin (g/L)	28 (24–31)
Hematocrit (%)	26 (24–28)
Outcomes	
Death in ICU	12 (30.0)
RRT at ICU discharge	17 (42.5)
Death in hospital	15 (37.5)
RRT at hospital discharge	4 (10.0)

Data are presented as median (interquartile range) for continuous measures, and *n* (%) for categorical measures.

CRRT, Continuous renal replacement therapy; ICU, Intensive care unit; RRT, Renal replacement therapy; SOFA, Sequential Organ Failure Assessment.

The total observation duration ranged from 13 to 90 h per patient. 27% of all single observations fell during exclusion periods (details in Figure 1); hence, 223,660 single observations (1243 h) were considered for analyses (details per patient in Supplementary Table S1).

### HPI alarms and HI

HPI was ≥ 85 in 33.4% of observations and MAP was <65 mmHg in 11.0%. Altogether, 65,801 HPI alarms and 18,388 HI (including 20 initiations of vasopressors, 31 increases of vasopressors, and 8 clinical signs of poor tolerance) were recorded. Distribution of events across patients was heterogeneous (Supplementary Tables S2, S3, and S4).

### Diagnostic accuracy

Overall, continuous HPI showed good discrimination for HI prediction within 15 min, with an AUC-ROC of 0.89 (Figure 2) and an AUPRC of 0.75 (Supplementary Figure S2). The number of TP, TN, FN, and FP is presented in Table 2. With the standard cut-off of 85 and the definitions described in the Methods section, HPI sensitivity was 99.7% (95% CI: 99.6 to 99.7), and its specificity was 81.6% (95% CI: 81.4 to 81.8). The NPV was also high at 99.9% (95% CI: 99.9 to 99.9), whereas the PPV was low at 54.7% (95% CI: 54.3 to 55.1). A total of 116 (0.6%) HI episodes were not preceded by an HPI alarm within the 15-min preceding window. Figure 3 shows the PPV of HPI alarms for predicting HI, along with the sensitivity, defined as the proportion of HI episodes correctly predicted by an HPI alarm. The positive likelihood ratio was 5.4.

**Figure 2:**
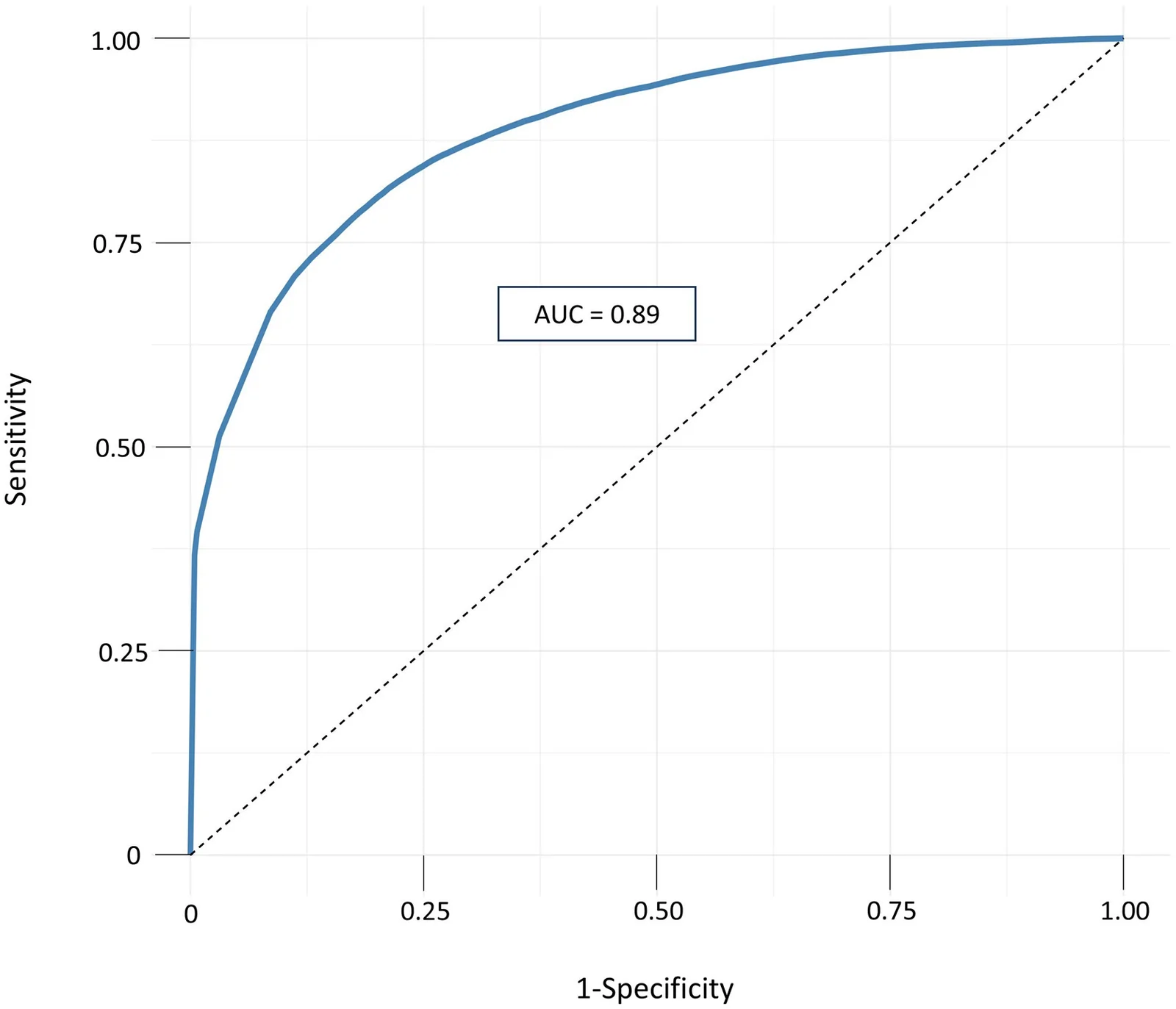
ROC curve of the hypotension prediction index to predict hemodynamic instability. ROC curve showing the performance of the hypotension prediction index in predicting episodes of hemodynamic instability in critically ill patients undergoing continuous renal replacement therapy. Hemodynamic instability was defined as MAP <65 mmHg for ≥1 min, vasopressor initiation or significant increase, or clinical signs of poor tolerance. The AUC quantifies the overall discriminative ability of HPI to predict HI within the next 1–15 min. AUC, Area under the curve; MAP, Mean arterial pressure; ROC, Receiver operating characteristic curve.

**Table 2:** Prediction of hemodynamic instability by HPI alarm.

Definitions	True positive	True negative	False negative	False positive	Delay alarm-event* (s, mean ± SD)
Cut-off for HPI alarm=85					
All events	35,973	132,105	116	29,814	103.2 ± 117.1
New events (Δ=60 min)†	33	993	141	114	258.9 ± 216.6
New events (Δ=3 min)†	283	11,884	508	640	245.9 ± 119.7
Cut-off for HPI alarm=75					
All events	38,299	122,373	70	38,216	112.6 ± 121.8
New events (Δ=60 min)†	23	958	152	110	239.2 ± 191.1
New events (Δ=3 min)†	247	11,136	539	693	274.4 ± 154.6

Number of true positive, true negative, false negative and false positive observed for different definitions of HPI alarms and HI episodes. A true positive is an HPI alarm followed by at least one HI within 15 min. A false positive is an HPI alarm not followed by an HI within 15 min. A false negative is an HI not preceded by any HPI alarm in the previous 15 min. A true negative is a time point with three consecutive values of HPI <85 and not followed by any HI within 15 min (in the new events analysis, to prevent artificial inflation of true negative counts, the analysis window was moved forward by a time interval equal to the event-free period after each true negative identification).

⁎ The mean of median delays observed for each patient between an alarm and the closest hemodynamic instability episode observed within 15 min.

† Pragmatic analysis considering only new events (Δ=duration of the event-free period before occurrence of an event). HPI, Hypotension prediction index; HI, Hemodynamic Instability; SD, Standard deviation.

**Figure 3:**
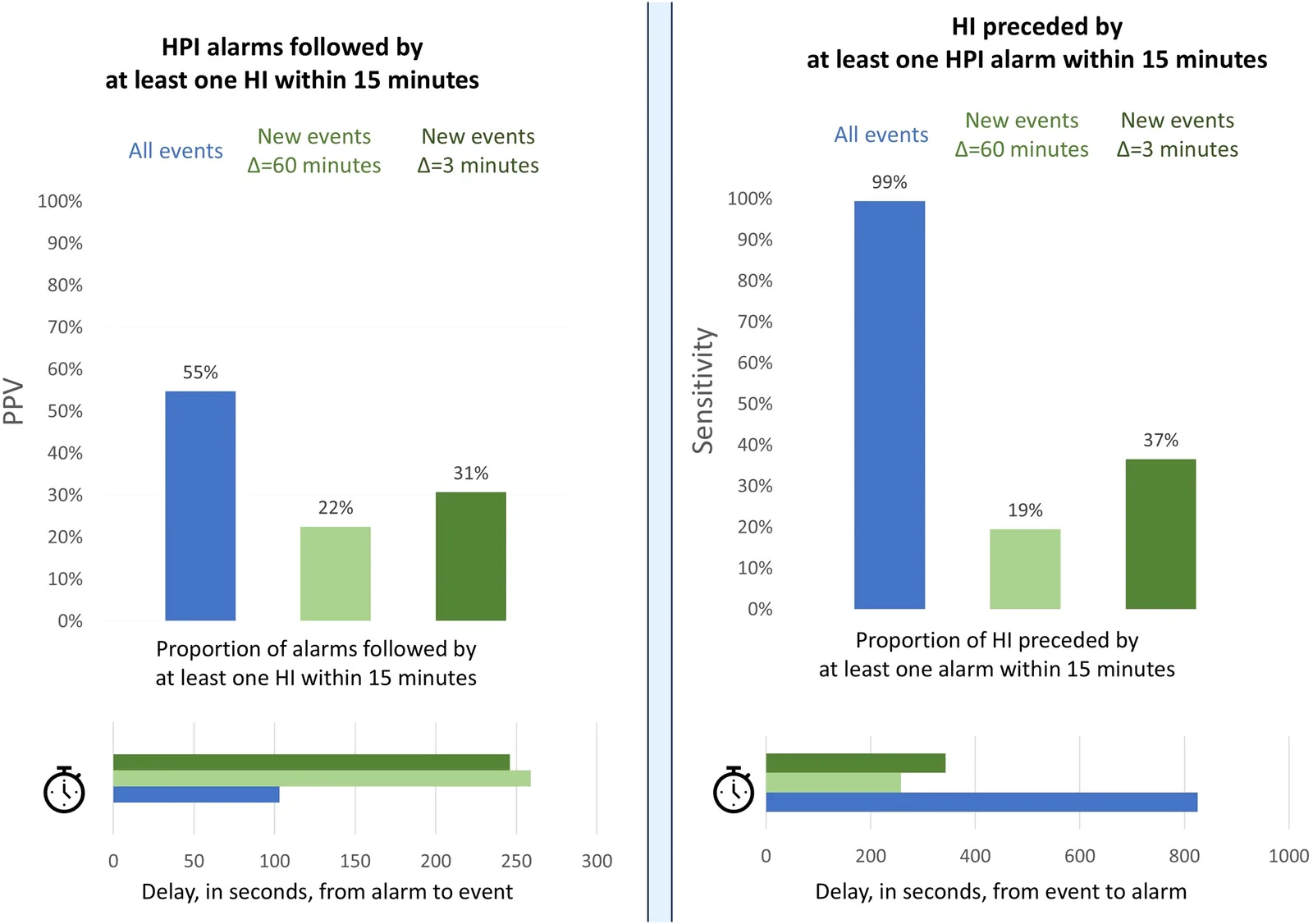
Proportion of HPI alarms followed by at least one hemodynamic instability episode and delay between events (left panel) and proportion of HI episodes preceded by at least one HPI alarm and delay between events (right panel). Dark blue: all events are considered in analyses (65,801 HPI alarms and 18,388 HI episodes). Light green: only new events occurring after a period free-of-events of 60 min (147 HPI alarms and 174 HI episodes). Dark green: only new events occurring after a period free-of-events of 3 min (924 HPI alarms and 802 HI episodes). The delay is the mean value of the median delays observed for each patient. HI, Hemodynamic instability; HPI, Hypotension prediction index; PPV, Positive predictive value.

These findings were confirmed by bootstrap analyses, performed to adjust for the varying importance of individual patients in the global dataset. In these analyses, PPV values were 57.8% ± 2.5%, sensitivity was 99.6% ± 0.1%, specificity was 82.5% ± 1.2%, and NPV was 99.8% ± 0.0%.

### Clinical utility

A strong association was observed between simultaneous HPI and MAP values (pseudo *R*^2^=0.98), and the density plot indicated that when HPI exceeds 85, the simultaneous MAP is already near or below 65 (Figure 4). Furthermore, the MAP concurrent with TP HPI alarms was (63.4 ± 3.8) mmHg, and only 2.1% (*n=*748) of values exceeded 70 mmHg. When considering MAP values temporally following HPI, the strength of the association between HPI and subsequent MAP decreased with the temporal gap but remained robust at a 15-min horizon (pseudo *R*^2^=0.73 for HPI predicting MAP at +15 min).

**Figure 4:**
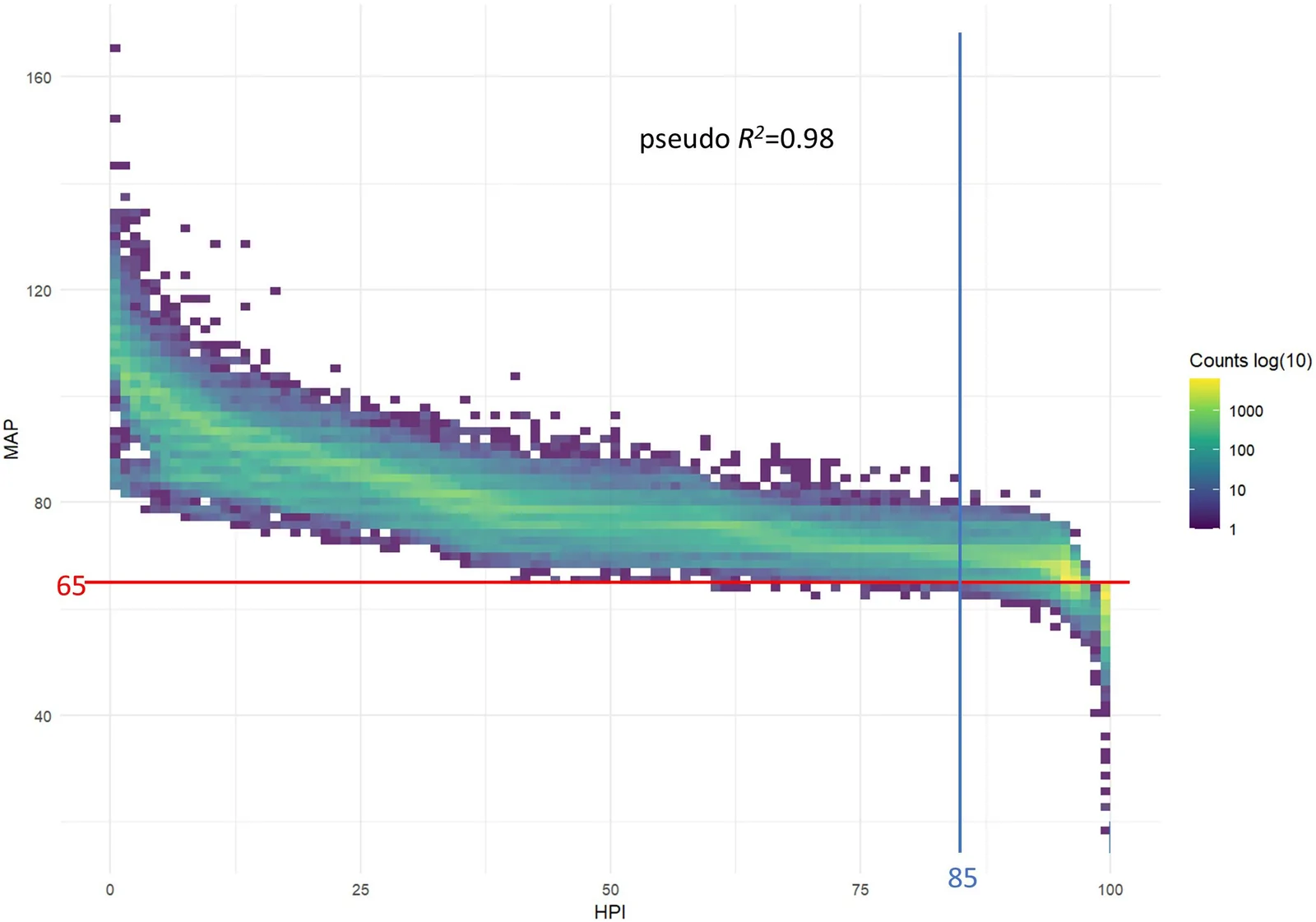
Density plot of simultaneous hypotension probability index (HPI) and mean arterial pressure (MAP) values. Each colored dot represents the log₁₀ number of simultaneous HPI–MAP recordings (dark purple=under 10 observations, yellow‐green=over 1000 observations). The horizontal red line marks the clinical hypotension threshold (MAP=65 mmHg), and the vertical blue line indicates the standard HPI alarm cut-off (HPI=85).

The mean delay between HPI alarm and the occurrence of an HI within 15 min was (103 ± 117) s.

The median number of alarms per hour across individuals was 41 (IQR: 5–72), of which 23 were false-positive alarms; the individual breakdown of alarms per hour is presented in Supplementary Table S5.

To account for the strong correlation between HPI and MAP and the prolonged duration of events, we conducted a pragmatic analysis focusing exclusively on new HPI alarms and new HI (see statistical analyses for definitions).

Considering new events preceded by a 60 min event-free-period, we identified 147 new HPI alarms and 174 new HI episodes. Of these, 33 (22.4%) new HPI alarms correctly predicted a new HI within 15 min and 34 (19.4%) of new HI were correctly predicted by a new HPI alarm in the previous 15 min (Figure 3). With this definition, the mean delay between the new HPI alarm and the new HI was (259 ± 217) s.

### Sensitivity analyses

We repeated our analyses considering new events with an event-free period of 3 min instead of 60 min. Here, we observed 924 new HPI alarms and 802 new HI. Of these, 284 new HPI alarms (30.7%) correctly predicted a new HI within the subsequent 15 min; conversely, 292 new HI episodes (36.5%) were preceded by at least one new HPI alarm within the prior 15-min window (Figure 3).

When considering 75 instead of 85 as a threshold to define an HPI alarm, we observed 76,515 alarms, 132 new alarms considering an event-free period of 60 min and 941 new alarms considering an event-free period of 3 min. Considering all values, 38,299 HPI alarms (50.1%) correctly predicted an HI, and 18,315 of HI episodes (99.6%) were correctly predicted. Considering new events with an event-free period of 60 min, 22 new HPI alarms (16.6%) correctly predicted a new HI within 15 min and, conversely, 23 of the new HI episodes (13.1%) were correctly predicted by a new HPI alarm occurring in the preceding 15 min. Considering new events with an event-free period of 3 min, 247 new HPI alarms (26.3%) correctly predicted a new HI within 15 min and, conversely, 261 of the new HI episodes (32.6%) were correctly predicted by a new HPI alarm occurring in the preceding 15 min. Altogether, lowering the HPI threshold from 85 to 75 slightly reduced the proportion of correct predictions (Supplementary Figure 3).

## Discussion

### Key findings

We conducted a prospective observational study to assess the discriminative properties and clinical utility of HPI to predict HI in critically ill patients undergoing CRRT with fluid removal. We found that, when all datapoints were considered, HPI showed good discrimination for HI diagnosis with an AUC ROC of 0.89 and HPI alarm had a high sensitivity and specificity. The AUPRC (0.75) was markedly higher than the baseline risk of HI (HI prevalence was 11.0%), indicating substantial enrichment of true events among high HPI values. However, we also found that HPI was above the alarm threshold more than a third of the time and that it was highly and more associated with simultaneous MAP than with future MAP, suggesting that HPI may not “predict” hypotension, but rather flag an event that is already happening at the cost of an important alarm burden. Hence, when only new events and new alarms were considered, HPI performance was much lower, with less than a quarter of alarms followed by events and less than one in five events preceded by an alarm. Sensitivity analyses using a lower threshold to define HPI alarm and a shorter duration to define new events confirmed these findings. Finally, regardless of the operational definition employed, the median time interval between HPI alarm activation and subsequent HI onset was approximately 2 min.

### Comparison with other studies

Previous studies conducted in the operating room reported conflicting results. Initial evaluations reported an excellent diagnostic accuracy of HPI to predict hypotension with sensitivity and specificity reaching up to 86%.^[^^16^^,^^25^^]^ However, these results were recently challenged and might have been subject to time trend and selection biases. Indeed, the HPI algorithm obviously integrates MAP in its prediction and might simply mirror its measurements. In addition, HPI might overestimate the risk of hypotension.^[^^26^^,^^27^^]^ Recently, Rellum et al.^[^^28^^]^ in a surgical setting, found that HPI alarms had a low PPV for hypotension (55.6% (IQR: 55.4–55.8)) and only preceded the event by 2.6 min. Despite being obtained in a different clinical context, our results are consistent with these studies.

In our dataset, the extremely large number of observations and their high-frequency may have introduced bias. Indeed, consecutive values of HPI are highly autocorrelated, and adjacent measures differ only minimally. Hence, an alarm (or an absence of alarm) typically persists over multiple consecutive timepoints. This temporal dependence between measures violates the assumption of independence underlying conventional diagnostic metrics. As a result, test metrics (sensitivity, PPV, specificity, and NPV) may be inflated when multiple alarms precede a single HI or when long periods without alarms occur before HI-free periods. In both cases, repeated counting may distort apparent test performance.

To overcome this issue, different strategies have been proposed without a consensus analytical framework. Most authors chose to shift the 20-min window forward in time after a TP, FP, or FN occurred and to count a maximum of one TN every 20 min. Others used a backward approach, starting from the event and looking whether it had been predicted or not.^[^^15^^,^^23^^,^[26], [27], [28]^]^ While this limits inflation, it does not fully reflect clinical practice, where a clinician may consider HPI value at any point in time. We have elected, in our main analysis, for TP, TN, and FP assessment, to use a forward-looking approach where the HPI alarm served as the reference point and subsequent HI were sought. To determine FN, on the other hand, we used a backward-looking approach where HI served as a reference point, and HPI alarms in the preceding 15 min were sought to assess for adequate prediction.

Then, recognizing that standard test metrics were not designed for continuous, autocorrelated monitoring data such as HPI, we completed our main analysis with a more pragmatic, clinically oriented assessment. This considered only new and independent events, giving more importance to the alarm-to-event delay, and reporting simple measures such as the proportion of events not predicted and the proportion of alarms not followed by an event. In our secondary analysis, we also chose to advance the observation window after each TN, consistent with prior studies, to further reduce multiple counting.

Our population is particular as it presents a higher incidence of hypotension compared to previous studies conducted in the ICU.^[^^29^^]^

### Implications for clinicians and policy makers

The extremely short delay between the alarm and the event, as well as the high proportion of alarms not followed by an event, limits the utility of a positive HPI alarm to guide diagnostic workups or therapeutic actions. The risk of such a low PPV is thus to increase the number of unnecessary or even detrimental diagnostic or therapeutic actions (such as fluid loading). Also, the added value of HPI compared to the readily available MAP value can be questioned, as the two values appear highly correlated. This highlights that good discrimination of a monitoring tool does not necessarily translate into clinical usefulness. Finally, the high number of alarms per hour (>41) may lead to alarm fatigue.

On the other hand, the observed high NPV suggests that HPI could be used to reassure the clinical team and encourage further fluid removal. Again, this is limited by the short delay between alarm and HI. Protocols designed to tailor the ultrafiltration rate based on HPI should be tested in prospective interventional trials.^[^^30^^]^

### Strengths and limitations

Our study has several strengths. First, this is the first to assess HPI’s performance in the challenging context of fluid removal during CRRT. This is thought to represent a near-experimental model where fluid removal might trigger a hypotensive event in otherwise stable patients.^[^^31^^]^ In addition, the ability to predict hypotension may be of particular relevance in this situation to guide fluid removal. Second, we performed a thorough evaluation of the tools’ performance, including >1000 h of recording in a large panel of critically ill patients, yielding precise estimates for the test diagnostic accuracy. Third, bootstrap analyses were performed to control for the different durations of observations per patient. Fourth, to mitigate bias associated with HPI and MAP autocorrelation, we considered only new alarms and events with 3 and 60 min free intervals. Fifth, in addition to real hypotension, we have included clinically relevant surrogates such as norepinephrine dose increase or clinical signs of poor tolerance to ultrafiltration in our event definition; this limits FNs associated with preventive clinicians’ measures.

On the other hand, our study also has limitations worth discussing. The small number of patients, their important heterogeneity, and the exclusion of patients in whom clinicians anticipated intolerance and therefore did not initiate net ultrafiltration may limit the generalizability of our results. However, the prolonged observation periods enabled the capture of a large number of events, ensuring adequate statistical power. When assessing the prediction ability of a technique with high frequency data in the context of low rate of event, true and FN calculation is challenging and lacks consensus. Because PPV is strongly influenced by event incidence, its value is inherently limited in the context of rare events such as HI. Thus, despite its high sensitivity and specificity, HPI yielded a large number of false alarms, reducing clinical utility and potentially contributing to alarm fatigue. Conversely, the NPV remained high, reflecting the reassuring absence of an impending HI when no alarm was triggered. We have elected to report on clinically relevant elements such as the rate of alarms followed by an event and the number of events preceded by an alarm. Finally, the reason for CRRT initiation was not collected.

## Conclusions

When all values are considered, HPI shows good discrimination to predict HI during CRRT with fluid removal. However, its clinical utility is limited by a short time-to-event delay, a high proportion of FPs, a significant number of new events not predicted, and the prolonged time spent with values ≥85, leading to alarm fatigue.

## CRediT authorship contribution statement

**Céline Monard:** Writing – review & editing, Writing – original draft, Investigation, Formal analysis, Data curation. **Clemence Ferlay:** Writing – review & editing, Investigation, Conceptualization. **Livia Whiting:** Writing – review & editing, Investigation, Conceptualization. **Bastien Trächsel:** Writing – review & editing, Formal analysis, Data curation. **Antoine Schneider:** Writing – review & editing, Writing – original draft, Validation, Supervision, Resources, Project administration, Methodology, Investigation, Funding acquisition, Formal analysis, Data curation, Conceptualization.

## Appendix Supplementary materials

Image, application 1
