# Supplementary material for: Hypotension prediction index to predict hemodynamic instability related to renal replacement therapy in critically ill patients: A prospective observational study
Source: J Intensive Med. 2026 May 9;6(5):475–83. doi: 10.1016/j.jointm.2026.03.002 (PMC13728361; doi:10.1016/j.jointm.2026.03.002)
Supplement: Supplementary file 1 [file mmc1.docx]

Supplementary File

**Using the HPI Warning System to Predict Hemodynamic Instability during Fluid Removal with CRRT in critically ill patients**

**Authors:** Céline Monard, Clemence Ferlay, Livia Whiting, Bastien Trächsel, Antoine Schneider

[Figure S1: Events and new events definitions	2](#_Toc225523563)

[Table S1- Breakdown of number and proportion of included observations per patient.	3](#_Toc225523564)

[Table S2: Breakdown of number and proportion of HPI values ≥85 per patient	4](#_Toc225523565)

[Table S3: Breakdown of number and proportion of alarms per patient	5](#_Toc225523566)

[Table S4: Breakdown of number and proportion of hemodynamic instability events per patient	6](#_Toc225523567)

[Figure S2: Precision recall curve of the Hypotension Prediction Index (HPI) to predict hemodynamic instability	7](#_Toc225523568)

[Table S5: Average number of alarms per hour of observation, for each patient	8](#_Toc225523569)

[Figure S3 : Proportion of HPI alarms followed by at least one hemodynamic instability (HI) episode and delay between events (left panel) and proportion of HI episodes preeceeded by at least one HPI alarm and delay between events (right panel) with a threshold for HPI alarm set at 75.	10](#_Toc225523570)

# Figure S1: Events and new events definitions

Hemodynamic instability (HI, blue circles) is diagnosed at a time point if the 2 previous and the considered MAP values are <65mmHg, vasopressors are initiated/increased (>5μg/min over 30 minutes) or clinical signs of hypoperfusion appear. An HPI alarm (Al, green circles) is diagnosed at a time point if the 2 previous and the considered HPI values are ≥85. An event-free period of 60 minutes has been determined to define independent events. Hence, a new hemodynamic instability is an HI preceded by an event-free period of duration >60 min. A new alarm is an HPI alarm preceded by an event-free period of duration >60 min.

MAP: mean arterial pressure, HI: hemodynamic instability, HPI: hypotension prediction index.

# Table S1- Breakdown of number and proportion of included observations per patient.

An observation was obtained every 20 seconds and consisted of a set of values including simultaneous HPI, MAP, and norepinephrine dose

| Patient | Total number of recorded observations | Excluded  (no fluid removal) | Excluded  (other reasons) | Analyzed for HPI |
| --- | --- | --- | --- | --- |
| 1 | 3,977 | 730 (18.3) | 444 (11.2) | **2,803 (70.5)** |
| 2 | 4,054 | 210 (5.2) | 259 (6.4) | **3,585 (88.6)** |
| 3 | 12,593 | 511 (4.1) | 1,720 (13.7) | **10,362 (82.3)** |
| 4 | 8,192 | 428 (5.2) | 841 (10.3) | **6,923 (84.5)** |
| 5 | 12,721 | 0 (0.0) | 827 (6.5) | **11,894 (93.5)** |
| 6 | 7,082 | 0 (0.0) | 2,674 (37.8) | **4,408 (62.2)** |
| 7 | 16,636 | 7,503 (45.1) | 1,150 (6.9) | **7,983 (47.9)** |
| 8 | 3,073 | 2,628 (85.6) | 215 (7.0) | **230 (7.5)** |
| 9 | 4,366 | 299 (6.9) | 1,046 (23.9) | **3,021 (69.2)** |
| 10 | 16,232 | 331 (2.0) | 2,318 (14.3) | **13,583 (83.8)** |
| 11 | 11,837 | 1,078 (9.1) | 4,069 (34.4) | **6,690 (56.5)** |
| 12 | 11,866 | 534 (4.5) | 4,240 (35.7) | **7,092 (59.8)** |
| 13 | 12,779 | 360 (2.8) | 113 (0.9) | **12,306 (96.3)** |
| 14 | 8,003 | 358 (4.5) | 246 (3.1) | **7,399 (92.4)** |
| 15 | 8,645 | 722 (8.3) | 4,063 (47.0) | **3,860 (44.6)** |
| 16 | 7,539 | 186 (2.5) | 1,079 (14.3) | **6,274 (83.2)** |
| 17 | 8,394 | 0 (0.0) | 1,242 (14.8) | **7,152 (85.2)** |
| 18 | 4,855 | 0 (0.0) | 264 (5.4) | **4,591 (94.2)** |
| 19 | 9,267 | 4,995 (53.9) | 553 (6.0) | **3,719 (40.1)** |
| 20 | 4,194 | 1,601 (38.2) | 641 (15.3) | **1,952 (46.6)** |
| 21 | 13,372 | 5,402 (40.4) | 640 (4.8) | **7,330 (54.8)** |
| 22 | 8,010 | 183 (2.3) | 1,410 (17.6) | **6,417 (80.1)** |
| 23 | 5,112 | 0 (0.0) | 174 (3.4) | **4,938 (96.6)** |
| 24 | 12,458 | 0 (0.0) | 974 (7.8) | **11,484 (92.2)** |
| 25 | 7,842 | 306 (3.9) | 847 (10.8) | **6,689 (85.2)** |
| 26 | 2,773 | 0 (0.0) | 1,456 (52.5) | **1,317 (47.5)** |
| 27 | 13,165 | 294 (2.2) | 4,223 (32.0) | **8,648 (65.7)** |
| 28 | 10,580 | 2,193 (20.7) | 1,896 (17.9) | **6,491 (61.4)** |
| 29 | 8,190 | 361 (4.4) | 3,688 (45.0) | **4,141 (50.5)** |
| 30 | 3,416 | 0 (0.0) | 180 (5.3) | **3,236 (94.7)** |
| 31 | 4,329 | 0 (0.0) | 38 (0.9) | **4,291 (99.1)** |
| 32 | 4,589 | 0 (0.0) | 994 (21.7) | **3,595 (78.4)** |
| 33 | 2,496 | 0 (0.0) | 1,258 (50.4) | **1,238 (49.7)** |
| 34 | Patient 34 withdrew consent | | | |
| 35 | 4,293 | 186 (4.3) | 34 (0.8) | **4,073 (95.1)** |
| 36 | 7,458 | 172 (2.3) | 134 (1.8) | **7,152 (95.9)** |
| 37 | 8,224 | 0 (0.0) | 53 (0.6) | **8,171 (99.4)** |
| 38 | 3,179 | 0 (0.0) | 328 (10.3) | **2,851 (89.6)** |
| 39 | 4,590 | 0 (0.0) | 3,918 (85.4) | **672 (14.6)** |
| 40 | 3,367 | 980 (29.1) | 170 (5.0) | **2,217 (65.8)** |
| 41 | 4,760 | 0 (0.0) | 1,878 (39.4) | **2,882 (60.5)** |
| Total | **308,508** | **32,551 (10.6)** | **52,297 (17.0)** | **223,660 (72.4)** |

# Table S2: Breakdown of number and proportion of HPI values ≥85 per patient

| Patient | Observations  with HPI <85, n (%) | Observations  with HPI (≥85), n (%) | Total |
| --- | --- | --- | --- |
| 1 | 699 (24.94%) | 2,104 (75.06%) | 2,803 |
| 2 | 2,487 (69.37%) | 1,098 (30.63%) | 3,585 |
| 3 | 8,886 (85.76%) | 1,476 (14.24%) | 10,362 |
| 4 | 6,923 (100.00%) | 0 (0.00%) | 6,923 |
| 5 | 11,136 (93.63%) | 758 (6.37%) | 11,894 |
| 6 | 4,408 (100.00%) | 0 (0.00%) | 4,408 |
| 7 | 4,144 (51.91%) | 3,839 (48.09%) | 7,983 |
| 8 | 230 (100.00%) | 0 (0.00%) | 230 |
| 9 | 140 (4.63%) | 2,881 (95.37%) | 3,021 |
| 10 | 9,988 (73.53%) | 3,595 (26.47%) | 13,583 |
| 11 | 439 (6.56%) | 6,251 (93.44%) | 6,69 |
| 12 | 3,753 (52.92%) | 3,339 (47.08%) | 7,092 |
| 13 | 8,551 (69.49%) | 3,755 (30.51%) | 12,306 |
| 14 | 6,506 (87.93%) | 893 (12.07%) | 7,399 |
| 15 | 2,899 (75.10%) | 961 (24.90%) | 3,86 |
| 16 | 3,533 (56.31%) | 2,741 (43.69%) | 6,274 |
| 17 | 4,638 (64.85%) | 2,514 (35.15%) | 7,152 |
| 18 | 83 (1.81%) | 4,508 (98.19%) | 4,591 |
| 19 | 2,351 (63.22%) | 1,368 (36.78%) | 3,719 |
| 20 | 1,034 (52.97%) | 918 (47.03%) | 1,952 |
| 21 | 2,086 (28.46%) | 5,244 (71.54%) | 7,33 |
| 22 | 4,986 (77.70%) | 1,431 (22.30%) | 6,417 |
| 23 | 328 (6.64%) | 4,61 (93.36%) | 4,938 |
| 24 | 11,41 (99.36%) | 74 (0.64%) | 11,484 |
| 25 | 6,324 (94.54%) | 365 (5.46%) | 6,689 |
| 26 | 1,316 (99.92%) | 1 (0.08%) | 1,317 |
| 27 | 3,178 (36.75%) | 5,47 (63.25%) | 8,648 |
| 28 | 2,509 (38.65%) | 3,982 (61.35%) | 6,491 |
| 29 | 2,654 (64.09%) | 1,487 (35.91%) | 4,141 |
| 30 | 2,527 (78.09%) | 709 (21.91%) | 3,236 |
| 31 | 3,094 (72.10%) | 1,197 (27.90%) | 4,291 |
| 32 | 1,378 (38.33%) | 2,217 (61.67%) | 3,595 |
| 33 | 1,178 (95.15%) | 60 (4.85%) | 1,238 |
| 35 | 2,022 (49.64%) | 2,051 (50.36%) | 4,073 |
| 36 | 5,11 (71.45%) | 2,042 (28.55%) | 7,152 |
| 37 | 7,542 (92.30%) | 629 (7.70%) | 8,171 |
| 38 | 2,851 (100.00%) | 0 (0.00%) | 2,851 |
| 39 | 641 (95.39%) | 31 (4.61%) | 672 |
| 40 | 2,196 (99.05%) | 21 (0.95%) | 2,217 |
| 41 | 2,881 (99.97%) | 1 (0.03%) | 2,882 |
| Total | **149,039 (66.64%)** | **74,621 (33.36%)** | **223,660** |

# Table S3: Breakdown of number and proportion of alarms per patient

| Patient | Alarms with 85 as a threshold  N (%) | Alarms with 75 as a threshold  n (%) | New alarms with 85 as a threshold and an event-free period of 60 minutes  n (%) | New alarms with 85 as a threshold and an event-free period of 3 minutes  n (%) | Total observations |
| --- | --- | --- | --- | --- | --- |
| 1 | 2,010 (71.7) | 2,084 (74.4) | 1 (0.0) | 16 (0.6) | 2,791 |
| 2 | 996 (27.8) | 1,295 (36.1) | 4 (0.1) | 12 (0.3) | 3,565 |
| 3 | 1,345 (13.0) | 1,717 (16.6) | 9 (0.1) | 22 (0.21) | 10,328 |
| 4 | 0 (0.0) | 0 (0.0) | 0 (0.0) | 0 (0.0) | 6,884 |
| 5 | 692 (5.8) | 785 (6.6) | 3 (0.0) | 7 (0.1) | 11,846 |
| 6 | 0 (0.0) | 0 (0.0) | 0 (0.0) | 0 (0.0) | 4,397 |
| 7 | 3,104 (38.9) | 3,867 (48.4) | 8 (0.1) | 48 (0.6) | 7,929 |
| 8 | 0 (0.0) | 0 (0.0) | 0 (0.0) | 0 (0.0) | 221 |
| 9 | 2,832 (93.7) | 2,876 (95.2) | 1 (0.0) | 2 (0.1) | 3,007 |
| 10 | 2,708 (19.9) | 3,803 (28.0) | 14 (0.1) | 95 (0.7) | 13,502 |
| 11 | 6,148 (91.9) | 6,285 (94.0) | 1 (0.0) | 16 (0.2) | 6,672 |
| 12 | 2,680 (37.8) | 3,405 (48.0) | 4 (0.1) | 67 (0.9) | 7,044 |
| 13 | 3,328 (27.0) | 3,895 (31.7) | 12 (0.1 | 43 (0.4) | 12,238 |
| 14 | 693 (9.4) | 937 (12.7) | 10 (0.1) | 39 (0.5) | 7,373 |
| 15 | 737 (19.1) | 958 (24.8) | 3 (0.1) | 16 (0.4) | 3,850 |
| 16 | 2,507 (40.0) | 2,886 (46.0) | 5 (0.1) | 27 (0.4) | 6,225 |
| 17 | 2,267 (31.7) | 2,508 (33.1) | 4 (0.1) | 18 (0.3) | 7,118 |
| 18 | 4,460 (97.2) | 4,483 (97.7) | 1 (0.0) | 4 (0.1) | 4,561 |
| 19 | 1,139 (30.6) | 1,278 (34.4) | 4 (0.1) | 35 (0.9) | 3,701 |
| 20 | 780 (40.0) | 998 (51.1) | 0 (0.0) | 20 (1.0) | 1,944 |
| 21 | 4,887 (66.7) | 5,476 (74.7) | 2 (0.0) | 45 (0.6) | 7,292 |
| 22 | 813 (12.7) | 1,245 (19.4) | 9 (0.1) | 52 (0.8) | 6,388 |
| 23 | 4,288 (86.8) | 4,455 (90.2) | 0 (0.0) | 13 (0.3) | 4,899 |
| 24 | 64 (0.6) | 94 (0.8) | 0 (0.0) | 0 (0.0) | 11,424 |
| 25 | 186 (2.8) | 317 (4.7) | 5 (0.1) | 32 (0.5) | 6,667 |
| 26 | 0 (0.0) | 3 (0.2) | 0 (0.0) | 0 (0.0) | 1,307 |
| 27 | 5,021 (58.1) | 6,026 (69.7) | 4 (0.1) | 47 (0.5) | 8,622 |
| 28 | 3,641 (56.1) | 4,211 (64.9) | 5 (0.1) | 43 (0.7) | 6,435 |
| 29 | 1,236 (29.9) | 1,433 (34.6) | 5 (0.1) | 41 (1.0) | 4,108 |
| 30 | 314 (9.7) | 493 (15.2) | 3 (0.1) | 25 (0.8) | 3,105 |
| 31 | 1,048 (24.4) | 1,303 (30,4) | 8 (0.2) | 33 (0.8) | 4,267 |
| 32 | 1,992 (55.4) | 2,124 (59.1) | 4 (0.1) | 18 (0.5) | 3,583 |
| 33 | 37 (3.0) | 144 (11.6) | 1 (0.1) | 3 (0.2) | 1,229 |
| 35 | 1,764 (43.3) | 2,324 (57.1) | 3 (0.1) | 31 (0.8) | 4,049 |
| 36 | 1,573 (22.0) | 2,027 (28.3) | 8 (0.1) | 40 (0.6) | 7,078 |
| 37 | 506 (6.2) | 733 (9.0) | 5 (0.1) | 12 (0.2) | 8,135 |
| 38 | 0 (0.0) | 0 (0) | 0 (0.0) | 0 (0.0) | 2,831 |
| 39 | 5 (0.7) | 35 (5.2) | 1 (0.2) | 2 (0.3) | 662 |
| 40 | 0 (0.0) | 27 (1.2) | 0 (0.0) | 0 (0.0) | 2,196 |
| 41 | 0 (0.0) | 0 (0.0) | 0 (0.0) | 0 (0.0) | 2,866 |
| Total | **65,801 (29.4)** | **76,530 (34.2)** | **147 (0.1)** | **924 (0.4)** | **222,339** |

# Table S4: Breakdown of number and proportion of hemodynamic instability events per patient

| Patient | Observations without HI, n (%) | Observations with HI  n (%) | New HI after an event-free period of 60 minutes  n (%) | New HI after an event-free period of 3 minutes  n (%) | Total observations |
| --- | --- | --- | --- | --- | --- |
| 1 | 1,674 (60.0) | 1,117 (40.0) | 1 (0.0) | 37 (1.3) | 2,791 |
| 2 | 3,561 (99.9) | 4 (0.1) | 1 (0.0) | 1 (0.0) | 3,565 |
| 3 | 10,140 (98.2) | 188 (1.8) | 5 (0.1) | 10 (0.1) | 10,328 |
| 4 | 6,883 (100.0) | 1 (0.0) | 0 (0.0) | 0 (0.0) | 6,884 |
| 5 | 11,641 (98.3) | 205 (1.7) | 1 (0.0) | 8 (0.1) | 11,846 |
| 6 | 4,397 (100.0) | 0 (0.0) | 0 (0.0) | 0 (0.0) | 4,397 |
| 7 | 7,310 (92.2) | 619 (7.8) | 10 (0.1) | 42 (0.5) | 7,929 |
| 8 | 221 (100.0) | 0 (0.0) | 0 (0.0) | 0 (0.0) | 221 |
| 9 | 2,101 (69.9) | 906 (30.1) | 3 (0.1) | 23 (0.8) | 3,007 |
| 10 | 13,177 (97.6) | 325 (2.4) | 20 (0.2) | 34 (0.3) | 13,502 |
| 11 | 4,991 (74.8) | 1,681 (25.2) | 7 (0.1) | 45 (0.7) | 6,672 |
| 12 | 5,674 (80.5) | 1,370 (19.5) | 7 (0.1) | 86 (1.2) | 7,044 |
| 13 | 11,668 (95.3) | 570 (4.7) | 11 (0.1) | 25 (0.2) | 12,238 |
| 14 | 7,100 (96.3) | 273 (3.7) | 6 (0.1) | 12 (0.2) | 7,373 |
| 15 | 3,849 (100.0) | 1 (0.0) | 1 (0.0) | 1 (0.0) | 3,850 |
| 16 | 5,254 (84.4) | 971 (15.6) | 4 (0.1) | 43 (0.7) | 6,225 |
| 17 | 6,959 (97.8) | 159 (2.2) | 5 (0.1) | 21 (0.3) | 7,118 |
| 18 | 2,221 (48.7) | 2,340 (51.3) | 4 (0.1) | 27 (0.6) | 4,561 |
| 19 | 2,948 (79.7) | 753 (20.3) | 6 (0.2) | 34 (0.9) | 3,701 |
| 20 | 1,437 (73.9) | 507 (26.1) | 3 (0.2) | 12 (0.7) | 1,944 |
| 21 | 6,060 (83.6) | 1,232 (16.9) | 10 (0.2) | 50 (0.7) | 7,292 |
| 22 | 6,291 (98.5) | 97 (1.5) | 8 (0 .1) | 17 (0.3) | 6,388 |
| 23 | 3,437 (70.2) | 1,462 (29.8) | 5 (0.1) | 49 (1.0) | 4,899 |
| 24 | 11,359 (99.4) | 65 (0.6) | 0 (0.0) | 1 (0.0) | 11,424 |
| 25 | 6,658 (99.9) | 9 (0.1) | 1 (0.0) | 1 (0.0) | 6,667 |
| 26 | 1,307 (100.0) | 0 (0.0) | 0 (0.0) | 0 (0.0) | 1,307 |
| 27 | 8,210 (95.2) | 412 (4.8) | 11 (0.1) | 33 (0.4) | 8,622 |
| 28 | 5,467 (85.0) | 968 (15.0) | 8 (0.1) | 41 (0.6) | 6,435 |
| 29 | 3,077 (74.9) | 1,031 (25.1) | 5 (0.1) | 43 (1.0) | 4,108 |
| 30 | 3,095 (99.7) | 10 (0.3) | 4 (0.1) | 7 (0.2) | 3,105 |
| 31 | 4,115 (96.4) | 152 (3.6) | 9 (0.2) | 20 (0.5) | 4,267 |
| 32 | 3,495 (97.5) | 88 (2.5) | 4 (0.1) | 15 (0.4) | 3,583 |
| 33 | 1,229 (100.0) | 0 (0.0) | 0 (0.0) | 0 (0.0) | 1,229 |
| 35 | 3,923 (96.9) | 126 (3.1) | 9 (0.2) | 24 (0.6) | 4,049 |
| 36 | 6,623 (93.6) | 455 (6.4) | 5 (0.1) | 25 (0.4) | 7,078 |
| 37 | 7,850 (96.5) | 285 (3.5) | 3 (0.1) | 9 (0.1) | 8,135 |
| 38 | 2,831 (100.0) | 0 (0.0) | 0 (0.0) | 0 (0.0) | 2,831 |
| 39 | 662 (100.0) | 0 (0.0) | 0 (0.0) | 0 (0.0) | 662 |
| 40 | 2,196 (100.0) | 0 (0.0) | 0 (0.0) | 0 (0.0) | 2,196 |
| 41 | 2,866 (100.0) | 0 (0.0) | 0 (0.0) | 0 (0.0) | 2,866 |
| Total | **203,957 (91.7)** | **18,388 (8.3)** | **177 (0.1)** | **802 (0.4)** | **222,339** |

# Figure S2: Precision recall curve of the Hypotension Prediction Index (HPI) to predict hemodynamic instability

Precision recall curve showing the performance of the Hypotension Prediction Index (HPI) in predicting episodes of hemodynamic instability (HI) in critically ill patients undergoing continuous renal replacement therapy. Hemodynamic instability was defined as MAP <65 mmHg for ≥1 minute, vasopressor initiation or significant increase, or clinical signs of poor tolerance. Precision (positive predictive value) is plotted againt recall (sensitivity) accross HPI thresholds (color scale) for predicting HI within the subsequent 1 to 15 minutes.

# Table S5: Average number of alarms per hour of observation, for each patient

| Patient | Number of hours of observation | Number of alarms (85 as a threshold)  per hours |
| --- | --- | --- |
| 1 | 15.57 | 129.08 |
| 2 | 19.92 | 50.01 |
| 3 | 57.57 | 23.36 |
| 4 | 38.46 | 0 |
| 5 | 66.08 | 10.47 |
| 6 | 24.49 | 0 |
| 7 | 44.35 | 69.99 |
| 8 | 1.28 | 0 |
| 9 | 16.78 | 168.74 |
| 10 | 75.46 | 35.89 |
| 11 | 37.17 | 165.42 |
| 12 | 39.40 | 68.02 |
| 13 | 68.37 | 48.68 |
| 14 | 41.11 | 16.86 |
| 15 | 21.44 | 34.37 |
| 16 | 34.86 | 71.93 |
| 17 | 39.73 | 57.06 |
| 18 | 25.51 | 174.86 |
| 19 | 20.66 | 55.13 |
| 20 | 10.84 | 71.93 |
| 21 | 40.72 | 120.01 |
| 22 | 35.65 | 22.81 |
| 23 | 27.43 | 156.31 |
| 24 | 63.80 | 1.00 |
| 25 | 37.16 | 5.01 |
| 26 | 7.32 | 0 |
| 27 | 48.04 | 104.51 |
| 28 | 36.06 | 100.97 |
| 29 | 23.01 | 53.73 |
| 30 | 17.98 | 17.47 |
| 31 | 23.84 | 43.96 |
| 32 | 19.97 | 99.74 |
| 33 | 6.88 | 5.38 |
| 35 | 22.63 | 77.96 |
| 36 | 39.73 | 39.59 |
| 37 | 45.39 | 11.15 |
| 38 | 15.84 | 0 |
| 39 | 3.73 | 1.34 |
| 40 | 12.32 | 0 |
| 41 | 16.01 | 0 |

Figure S3 : Proportion of HPI alarms followed by at least one hemodynamic instability (HI) episode and delay between events (left panel) and proportion of HI episodes preeceeded by at least one HPI alarm and delay between events (right panel) with a threshold for HPI alarm set at 75.

In dark blue results when all events are considered in analyses (76 515 HPI alarms and 18 385 HI episodes). In light green, when only new events occurring after a period free-of-events of 60 minutes are considered (132 HPI alarms and 175 HI episodes). In dark green, when only new events occurring after a period free-of-events of 3 minutes are considered (940 HPI alarms and 800 HI episodes).

The delays presented are the mean (SD) of the median times observed per patients.
